# Mode-Independent and Mode-Interactive Failure Criteria for Unidirectional Composites Based on Strain Energy Density

**DOI:** 10.3390/polym12122813

**Published:** 2020-11-27

**Authors:** Nian Li, Cheng Ju

**Affiliations:** College of Mechanical and Power Engineering, Nanjing Tech University, Nanjing 211816, China; jcbangbangda@163.com

**Keywords:** composites, failure criterion, strength, strain energy density, failure mode interaction

## Abstract

The strain energy released plays a crucial role in generating macroscopic failure in unidirectional (UD) composites. This paper proposes two new strain energy-based failure criteria, regarding fiber-dominated and matrix-dominated failure mode as independent and interactive, respectively. The failure expression is formulated based on rigorous mathematical deducing, accompanied by physical interpretation. Based on the lack of experimentally feasible multi-axial strengths, a predefined assumption of infinite strength under bi-axial and tri-axial compressive stress provides the possibility for determining all coefficients only by using conventional uniaxial strengths. The failure envelopes predicted by the proposed criteria have been validated against experimental results under biaxial, off-axis and tri-axial loading cases. A better agreement with physical reality was achieved by the failure mode-interactive criterion, suggesting a wide range of applicability.

## 1. Introduction

Failure theories, which can be classified as either being macroscopic or microscopic, have been an utmost crucial issue in scientific research and even more so in engineering practice since the widespread application in advanced composites. Through the series of World Wide Failure Exercises (WWFEs) spanning the past two decades [1,2,3,4], the state-of-the-art has been well-reflected in an objective manner. Macroscopic failure criteria, which are widely used in engineering practice, are classified into three groups. The criteria in the first group predict failure status by directly comparing stresses (or strains) with respective strengths. The representatives are the maximum stress and the maximum strain criteria.

Since the above strength limit criteria neglect interactions of stress or strain components, failure theories of the second group are proposed to consider full stress interaction by employing a single quadratic or higher-order polynomial function of all potential stresses. Tsai and his co-workers originally proposed one of the most famous criteria of a single expression, i.e., the Tsai–Wu failure criterion [5]. Although the Tsai–Wu criterion has achieved great success over the past decades, it has been subjected to criticisms for being non-phenomenological or empirical [6]. These criteria of single expression fundamentally combine distinctive fracture mechanisms occurring within unidirectional (UD) composite materials, therefore, the debate on rationalism and robustness for failure predictions is to continue due to different mechanisms found at the microscale [7]. Moreover, such criteria are unable to distinguish internal failure modes that may cause difficulties in the subsequent analysis of failure evolution.

The third group refers to phenomenological failure criteria that are based on physical aspects of fracture. The Hashin failure theory [8,9] made a significant contribution to the formulation of composite failure criteria by employing a solid physical basis instead of purely empirical curve fitting. It initially separated different modes of failure by defining corresponding stress invariants. The classification regarding the failure of fibre reinforced plastic (FRP) composites (i.e., matrix cracking in tension and compression and fiber damage in tension and compression) is usually adopted and has inspired, or laid a basis for follow-up studies [10]. Despite extensive selection in engineering due to its simplicity of concept, Hashin’s work could not present a reasonable interpretation of the shear fracture impeding influence induced by a moderate transverse compressive stress [11]. Moreover, some inconceivable results, from the physical point of view, may be gained since some arbitrary measures were taken, as discussed in [12]. Following Mohr’s fracture hypothesis stating that failure would be exclusively caused by the stresses acting on the fracture plane, Puck and Schurmann [13] introduced the concept of the fracture plane into their phenomenological failure model. Puck’s criterion not only predicts the stress level leading to crack initiation but also provides the results of crack direction. It was ranked highly among all the nineteen participants in the WWFEs, and therefore, was suggested by organizers [2]. A further modification, with high relevance to Puck’s theory, was performed by incorporating in situ effects, shear nonlinearity and fiber kinking mechanisms [14,15]. However, additional employment of artificially defined parameters, e.g., inclination parameters, may cause disputes over the determination of their specific values [16].

So far, the majority of current phenomenological failure criteria for UD composites are constructed based on stresses/strains, e.g., stress invariants [9,17,18,19] or stresses acting on the fracture plane [11,13,14,15]. Nevertheless, despite remarkable success achieved and convincing physical meaning in the definition of failure-induced stress, it should be emphasized that the choice of quadratics is not based on physical reasoning but curve fitting considerations [9]. There has been an issue of the failure theories which has never been thoroughly investigated, and that is the physical interpretation of the order of failure equations. The only explanation provided is that quadratic polynomials can fit test data well, indicating no need to employ cubic or higher approximations. The authors would argue the rationality existing in the explanation and the form as these criteria are proposed. Even though failure theories fall in the category of phenomenological approaches, it should be clarified that this assumption remains empirical or artificial, no matter how rational the subsequent deduction is in the formulation of the criteria. Within the theoretical framework constructed by the stress invariant-based approach, it hardly seems capable to “physically” explain why cubic or higher approximations are not employed. It seems feasible to formulate a strain energy density criterion due to the quadratic nature of stress-energy density forms.

The macroscopic material failure, from a thermodynamic point of view, is the final consequence of an energy-driven destabilization process and is associated with the collective disruption of atomic bonds that is driven by the potential energy stored in the atomic bonds. In a mechanical system, a universal failure criterion at the macroscopic scale can be defined by a specific elastic strain energy density with its threshold value [20]. Efforts have been made to investigate the fracture behaviors of isotropic materials by using the energy concept. However, if the attention is shift to UD fiber-reinforcement composite materials, quite limited literature has been reported based on the strain energy release. The Tsai–Hill criterion is the extension of the classical von Mises yield condition to orthotropic materials [21]. The main deficiency is that it cannot identify the difference of strengths in tension and compression. Wolfe et al. [22,23] developed a strain energy-based failure model to predict failure behaviors of composites under multi-axial loadings, taking into account the effect of hydrostatic stress. But there would be no experimentally feasible scheme to measure certain parameters, which are introduced in their criterion to define the shape of the failure surface in strain energy space.

For the existing failure criteria that divide composite failure into fiber-dominated and matrix-dominated damage modes, conventionally most of them (e.g., the Hashin’s criterion and Puck’s criterion) accept the hypothesis that fiber fracture and matrix cracking may be mutually independent. This hypothesis is proposed based on the fact that the potential fracture planes regarding matrix and fiber failure are perpendicular, but it has been questioned by a series of experimental evidence [24]. This phenomenon is attributed to the mutual influence of localized micro-damages. Taking fiber failure, for example, it should be noted that micro-fractures of some fiber elements have already occurred before the lamina reaches its macroscopic strength. The existence of microfiber fractures will induce micro-cracks in the neighboring matrix and debonding of fiber–matrix interfaces. Besides, the ability to resist fiber failure will be weakened due to the effects of defects. Even under transverse stress, micro-cracks, initiating in the region of defects, may propagate along the fibers. To establish a more reasonable criterion at the macroscale, the interactions of micro-damages are required to be characterized from a macroscopic perspective. Hence Puck used a degradation factor to consider the weakening effect. The major drawback, however, is the lack of reliable experimental or mathematical methods to obtain the specific value of that factor.

The objective of the present paper was to propose new three-dimensional failure criteria for UD composites from a strain energy release point of view. The formulation of the failure expression is logically deduced based on physical interpretation, rather than through curve fitting considerations made by stress invariant-based criteria (e.g., Hashin’s criterion). Also, a mode-interactive model is further proposed to characterize the micro-interactions of matrix-dominated and fiber-dominated failure modes in the view of the macro level. For both mode-independent and mode-interactive criteria, all parameters required can be determined by conventional strength properties at the UD-lamina level, without making any assumption sometimes arguable. Finally, the present failure criteria are validated and compared, in terms of failure envelopes, by measurements in biaxial, off-axis tension and tri-axial experiments.

## 2. Failure Mode-Independent Criterion Based on Strain Energy Release

The thermodynamics of irreversible processes, regarding energy conversion as an essential physical process, is a rational framework that can be applied to formulate failure criterion. Neglecting thermal effects, the mechanical work of the external load, *W*, is converted to the elastic strain energy $U_{e}$ and the dissipated energy $U_{d}$ in a closed system. According to the first law of thermodynamics, it assumes

(1) $$W=U_{e}+U_{d}$$

where irreversible $U_{d}$ denotes the plastic deformation and the internal damage induced by micro-cracks or defects inside a material element, while $U_{e}$ is releasable and reversible energy. When a material is subjected to the external load, some fraction of the mechanical work imposed on the UD composite is converted into the dissipated energy, which leads to a progressive deterioration in the cohesion of microscopic structures and corresponds to a nonlinear stress–strain behavior at the macro scale. Since material failure at different levels has different physical meanings, it should be noted that such degradation in mechanical properties of micro material elements does not necessarily imply a material failure on a macroscopic level. Taking uniform tri-axial compression for example, despite the property deterioration caused by micro damages at the microscopic level, a UD composite may not fail due to the pressure effect. Moreover, given that the intrinsic dissipation during a material brittle failure is negligible, a specified elastic strain energy is suggested to macroscopically represent a failure of using the energy density concept to define a universal macroscopic failure criterion [20]. For a UD composite under a general stress state $\sigma_{i}$ in a Cartesian coordinate, its total elastic strain energy is defined as

(2) $$U_{e}=\frac{1}{2}\sigma_{i}\varepsilon_{i}\left(i=1,2,\cdots ,6\right)$$

where $\varepsilon_{i}$ is the elastic strain. The Cartesian coordinate system is defined as follows: 1-direction corresponds to the fiber direction, 2-direction lies perpendicular to the fiber direction and 3-direction goes along the thickness-direction of the single layer. Given transverse isotropy that is usually sufficient to characterize behaviors of UD composites, Equation (2) can be further written as

(3) $$U_{e}=\frac{S_{11}}{2}\sigma_{11}^{2}+\frac{S_{22}}{2}{\left(\sigma_{22}+\sigma_{33}\right)}^{2}+S_{12}\sigma_{11}\left(\sigma_{22}+\sigma_{33}\right)+\frac{S_{44}}{2}\left(\tau_{23}^{2}-\sigma_{22}\sigma_{33}\right)+\frac{S_{66}}{2}\left(\tau_{12}^{2}+\tau_{13}^{2}\right)$$

where $S_{ij}(i,j=1,2,\cdots ,6)$ represent the components of the compliance matrix for a transverse isotropic material.

As long as the stored strain energy $U_{e}$ reaches a critical value $G_{c}$, it will be suddenly released, resulting in macroscopic failure. Specifically for brittle material, of which failure is independent of deformation history, the criterion may be generalized as

(4) $$U_{e}\left(\sigma_{ij}\right)=G_{c}$$

If the critical strain energy $G_{c}$ is assumed to be constant, a simple criterion can be constructed by substituting Equation (3) into Equation (4). That is

(5) $$\frac{S_{11}}{2G_{c}}\sigma_{11}^{2}+\frac{S_{22}}{2G_{c}}{\left(\sigma_{22}+\sigma_{33}\right)}^{2}+\frac{S_{12}}{G_{c}}\sigma_{11}\left(\sigma_{22}+\sigma_{33}\right)+\frac{S_{44}}{2G_{c}}\left(\tau_{23}^{2}-\sigma_{22}\sigma_{33}\right)+\frac{S_{66}}{2G_{c}}\left(\tau_{12}^{2}+\tau_{13}^{2}\right)=1$$

It is not surprising that Equation (5) reproduces a standard stress form of the classical Tsai–Hill failure criterion [21], since the Tsai–Hill criterion is the extension of the distortion strain energy-based von Mises yield condition to orthotropic materials. Nevertheless, such an assumption indicates the identical tensile and compressive nature of UD composite failure. Therefore, in principle, the present form of Equation (5) could only predict macroscopic failure of the composite material with exactly the same released strain energy or strength under tension and compression. Another main irrationality of this assumption may be the combination of fundamental fracture mechanisms that are distinct. The critical energy release corresponding to the fiber fracture (e.g., uniaxial tension along the longitudinal direction) is different from that corresponding to the matrix cracking (e.g., pure transverse shear). The above analysis shows that the simple assumption of $G_{c}$ as a constant is improper, the physical meanings underlying is not in satisfying agreement with the observation of actual composite failure mechanisms. As a result, in an attempt to provide reasonably rational physical explanations, the specific form of $G_{c}$ should meet the following conditions:

(a) In contrast to isotropic materials, the heterogeneous nature of UD fiber reinforcement composites indicates that there are two primary failure modes on the lamina level, i.e., longitudinal, fiber-dominated failure mode and matrix-dominated failure mode. So the critical energy release corresponding to different failure modes naturally comes different.

(b) The release of strain energy stored in composite materials is related to tension stress and compression stress, whereas pure shear stresses with opposite orientation play an identical role in failure formation, namely having the same critical strain energy.

(c) It seems impossible to neglect the effect of the hydrostatic stress on mechanical behavior, as polymers exhibit clear sensitivity to hydrostatic pressure. Experimental investigation on the effects of hydrostatic pressure has found that the transverse compression modulus increased markedly with pressure, while a slight increase appeared in the longitudinal modulus [25]. The phenomenon suggests that the volume change associated with hydrostatic stress may significantly affect the strain energy corresponding to transverse matrix failure modes.

As a consequence, a failure criterion is to be formulated from the viewpoint of energy, to distinguish between longitudinal, fiber-dominated failure mechanisms and transverse, matrix-dominated failure mechanisms on the lamina level. In general, it is appropriate to use four different formulas to describe these two primary modes induced by tension and compression.

### 2.1. Fibre Failure Mode

For the fiber failure mode, the failure plane is about the 2–3 plane [26]. The strain energy, $U_{e}^{\mathrm{ft},\mathrm{fc}}$, producing this type of failure may be correlated with $\sigma_{11}$, $\tau_{12}$ and $\tau_{13}$, resulting in the fiber-controlled failure criterion as

(6) $$U_{e}^{i}=\frac{S_{11}^{\mathrm{int}}}{2g(I_{1})}\sigma_{11}^{2}+\frac{S_{12}^{\mathrm{int}}}{g(I_{1})}\sigma_{11}\left(\sigma_{22}+\sigma_{33}\right)+\frac{S_{66}^{\mathrm{int}}}{2g(I_{1})}\left(\tau_{12}^{2}+\tau_{13}^{2}\right)=G_{c}^{i}\;\;\;\left(i=\mathrm{ft},\mathrm{fc}\right)$$

where $I_{1}$ ($I_{1}=\sigma_{11}$) is the first stress invariant with respect to the preferred direction coinciding with the 1-direction. The superscript ‘int’ denotes the initial status with $I_{1}=0$. $g(I_{1})$ is the functional relation for the initial longitudinal elastic modulus, $E_{11}^{\mathrm{int}}$, (where $I_{1}=0$) and $E_{11}^{\mathrm{hyd}}$ under hydrostatic stress (where $I_{1}\neq 0$). Tensile fiber failure and compressive fiber failure are denoted by superscripts i=ft and i=fc, respectively. Rearranging Equation (6) turns into

(7) $$U_{e}^{i}=\frac{S_{11}}{2}\sigma_{11}^{2}+S_{12}\sigma_{11}\left(\sigma_{22}+\sigma_{33}\right)+\frac{S_{66}}{2}\left(\tau_{12}^{2}+\tau_{13}^{2}\right)=G_{c}^{i}\;\left(I_{1}\right)\;\;\;\left(i=\mathrm{ft},\mathrm{fc}\right)$$

where the superscript ‘int’ is neglected for the sake of simplification. According to the experimental observation that longitudinal elastic modulus is insensitive to hydrostatic stress, i.e., $g(I_{1})=1$, the critical strain energy related to fiber tensile and compressive failure is approximately constant, namely

(8) $$G_{c}^{i}\left(I_{1}\right)=c_{i}$$

By applying pure longitudinal shear strength, $S_{L}$, to Equation (7), it follows

(9) $$c_{\mathrm{ft}}=c_{\mathrm{fc}}=\frac{S_{66}}{2}S_{L}$$

Utilizing failure data of uniaxial stress state $\sigma_{11}$ in Equation (7) results in

(10) $$X_{T}=\sqrt{\frac{S_{66}}{S_{11}}S_{L}},\;\;X_{C}=-\sqrt{\frac{S_{66}}{S_{11}}S_{L}}$$

where $X_{T}$ and $X_{C}$ are tensile and compressive failure stress in the fiber direction, respectively. Contradiction, however, emerges in most cases where the magnitude of $X_{T}$ is not equal to but higher than that of $X_{C}$. Thus rationally, the term of shear stress $\tau_{12}$ and $\tau_{13}$ is demonstrated to be not in the criterion regarding fiber failure. Equation (7) is then degenerated into

(11) $$U_{e}^{i}=\frac{S_{11}}{2}\sigma_{11}^{2}+S_{12}\sigma_{11}\left(\sigma_{22}+\sigma_{33}\right)=G_{c}^{i}\left(I_{1}\right)\;\;\;\left(i=\mathrm{ft},\mathrm{fc}\right)$$

This conclusion is not only correct mathematically but has physical meanings as well. It can be explained that in the case of pure longitudinal shear ($\tau_{12}$ or $\tau_{13}$), the strain energy required for shearing off fibers is much higher than shearing off of matrix. In other words, the energy stored in composite materials would be easier to release in the way of matrix cracking. Many micromechanical analyses [27,28,29] have also reported that the longitudinal shear loading would cause matrix cracking or fiber–matrix debonding at the 1-2 or 1-3 plane rather than shear-driven fiber breakage on the 2–3 plane. In addition, this inference is proved through the conclusions of Atas’s [30] and Tserpes’s work [31]. Both pieces of research have shown that fiber damage would be typically overestimated, once the conventional shear strength is applied in shear stress terms of the Hashin-type fiber tensile failure criterion. Hence, the energy-based criterion for fiber failure can be finally constructed in a stress type as fiber tension failure for $I_{1}\geq 0$ (FFT)

(12) $$F_{11}^{\mathrm{ft}}\sigma_{11}^{2}+F_{12}^{\mathrm{ft}}\sigma_{11}\left(\sigma_{22}+\sigma_{33}\right)=1$$

and fiber compression failure for $I_{1}<0$ (FFC)

(13) $$F_{11}^{\mathrm{fc}}\sigma_{11}^{2}+F_{12}^{\mathrm{fc}}\sigma_{11}\left(\sigma_{22}+\sigma_{33}\right)=1$$

with

(14) $$F_{11}^{i}=\frac{S_{11}}{2c_{i}},\;\;\;F_{12}^{i}=\frac{S_{12}}{c_{i}}$$

where the coefficients, $F_{11}^{i}(i=\mathrm{ft},\mathrm{fc})$, can be determined directly from conventional tensile and compressive strengths of the material along fibers as

(15) $$F_{11}^{\mathrm{ft}}=\frac{1}{X_{T}^{2}},\;\;\;F_{11}^{\mathrm{fc}}=\frac{1}{X_{C}^{2}}$$

The attempt of determining $F_{12}^{i}(i=\mathrm{ft},\mathrm{fc})$ means the inevitable consideration of multi-axial stress states, as the coefficients characterize a Poisson’s effect (reflected by the compliance matrix component $S_{12}$) and fiber compression failure for $\sigma_{11}$ and transverse stresses ($\sigma_{22}$ and/or $\sigma_{33}$). Given the difficulties in conducting this type of experiment and the lack of a standard experimental method, a case of tri-axial compression ($\sigma_{11}=\sigma_{22}=\sigma_{33}=-\sigma^{*}$) is considered. A logical outcome is deduced from the basic assumption supposing much higher strength that can be taken as infinite for mathematical convenience (i.e., $\sigma^{*}\to \infty$). Substituting this stress state into Equation (13)

(16) $$F_{12}^{\mathrm{fc}}=-\frac{F_{11}^{\mathrm{fc}}}{2}+\frac{1}{2{\left(\sigma^{*}\right)}^{2}}\approx -\frac{F_{11}^{\mathrm{fc}}}{2}=-\frac{1}{2X_{C}^{2}}$$

On the other hand, by rearranging Equation (14), one obtains

(17) $$\frac{F_{11}^{\mathrm{ft}}}{F_{12}^{\mathrm{ft}}}=\frac{F_{11}^{\mathrm{fc}}}{F_{12}^{\mathrm{fc}}}$$

Thus, $F_{12}^{\mathrm{ft}}$ can be approximated as

(18) $$F_{12}^{\mathrm{ft}}=-\frac{1}{2X_{T}^{2}}$$

### 2.2. Matrix Failure Mode

Similarly, the strain energy corresponding to matrix failure, $U_{e}^{m}$, would be formulated in terms of the stresses $\sigma_{22}$, $\sigma_{33}$, $\tau_{12}$, $\tau_{13}$ and $\tau_{23}$. Similarly, the matrix-controlled failure criterion is expressed of the general form

(19) $$\begin{array}{ll} U_{e}^{j} & =\frac{S_{22}}{2}{\left(\sigma_{22}+\sigma_{33}\right)}^{2}+S_{12}\sigma_{11}\left(\sigma_{22}+\sigma_{33}\right)+\frac{S_{44}}{2}\left(\tau_{23}^{2}-\sigma_{22}\sigma_{33}\right)+\frac{S_{66}}{2}\left(\tau_{12}^{2}+\tau_{13}^{2}\right)\\ & =G_{c}^{j}(I_{2})\;\;\;(j=\mathrm{mt},\mathrm{mc}) \end{array}$$

where $I_{2}$ ($I_{2}=\sigma_{22}+\sigma_{33}$) is the second stress invariant representing a volume change of the UD composite material caused by transverse normal stresses. Superscripts j=mt and j=mc distinguish tensile matrix failure with compressive matrix failure. It has been pointed out previously that hydrostatic stresses affect transverse elastic moduli significantly. So the critical energy $G_{c}^{j}$, as a function of $I_{2}$, could be derived by using a second-order approximation of Taylor’s expansion. It has

(20) $$G_{c}^{j}=a_{j}{\left(\sigma_{22}+\sigma_{33}\right)}^{2}+b_{j}\left(\sigma_{22}+\sigma_{33}\right)+c_{j}$$

Substituting Equation (20) into Equation (19) gives matrix tension failure for $I_{2}\geq 0$ (MFT)

(21) $$\begin{array}{c} F_{22}^{\mathrm{mt}}{\left(\sigma_{22}+\sigma_{33}\right)}^{2}+F_{2}^{\mathrm{mt}}\left(\sigma_{22}+\sigma_{33}\right)+F_{12}^{\mathrm{mt}}\sigma_{11}\left(\sigma_{22}+\sigma_{33}\right)\\ +F_{44}^{\mathrm{mt}}\left(\tau_{23}^{2}-\sigma_{22}\sigma_{33}\right)+F_{66}^{\mathrm{mt}}\left(\tau_{12}^{2}+\tau_{13}^{2}\right)=1 \end{array}$$

and matrix compression failure for $I_{2}<0$ (MFC)

(22) $$\begin{array}{c} F_{22}^{\mathrm{mc}}{\left(\sigma_{22}+\sigma_{33}\right)}^{2}+F_{2}^{\mathrm{mc}}\left(\sigma_{22}+\sigma_{33}\right)+F_{12}^{\mathrm{mc}}\sigma_{11}\left(\sigma_{22}+\sigma_{33}\right)\\ +F_{44}^{\mathrm{mc}}\left(\tau_{23}^{2}-\sigma_{22}\sigma_{33}\right)+F_{66}^{\mathrm{mc}}\left(\tau_{12}^{2}+\tau_{13}^{2}\right)=1 \end{array}$$

with

(23) $$F_{22}^{j}=\frac{S_{11}-2a_{j}}{2c_{j}},\;F_{2}^{j}=-\frac{b_{j}}{c_{j}},\;F_{12}^{j}=\frac{S_{12}}{2c_{j}},\;F_{44}^{j}=\frac{S_{44}}{2c_{j}},\;F_{66}^{j}=\frac{S_{66}}{2c_{j}}$$

Pure longitudinal or transverse shear stress ($S_{L}$ or $S_{T}$), in absence of all other stresses, is taken into account first by application of Equations (21) and (22)

(24) $$F_{44}^{\mathrm{mt}}=F_{44}^{\mathrm{mc}}=\frac{1}{S_{T}^{2}},\;F_{66}^{\mathrm{mt}}=F_{66}^{\mathrm{mc}}=\frac{1}{S_{L}^{2}}$$

For MFC, the available simple information is $\sigma_{22}=-Y_{C}$ for uniaxial transverse compression. The following equation can be deduced from the above stress state

(25) $$F_{22}^{\mathrm{mc}}Y_{C}^{2}-F_{2}^{\mathrm{mc}}Y_{C}=1$$

To achieve additional information, uniform biaxial compressive condition $\sigma_{22}=\sigma_{33}=-Y_{\mathrm{Cbi}}$ is imposed as a supplementary, leading to

(26) $$4F_{22}^{\mathrm{mc}}Y_{\mathrm{Cbi}}^{2}-2F_{2}^{\mathrm{mc}}Y_{\mathrm{Cbi}}-F_{44}^{\mathrm{mc}}Y_{\mathrm{Cbi}}^{2}=1$$

Then coefficients $F_{22}^{\mathrm{mc}}$ and $F_{2}^{\mathrm{mc}}$ are solved by combining Equation (25) and Equation (26)

(27) $$F_{2}^{\mathrm{mc}}=-\frac{\frac{1}{Y_{C}^{2}}\left(\frac{Y_{C}^{2}}{4Y_{\mathrm{Cbi}}^{2}}-1\right)+\frac{1}{4S_{T}^{2}}}{\frac{1}{Y_{C}}\left(\frac{Y_{C}}{2Y_{\mathrm{Cbi}}}-1\right)}$$

(28) $$F_{22}^{\mathrm{mc}}=\frac{1}{Y_{C}^{2}}+\frac{F_{2}^{\mathrm{mc}}}{Y_{C}}$$

However, there is no standard experimental procedure to measure biaxial compressive strength $Y_{\mathrm{Cbi}}$ at present. Therefore for the convenience of application, it is assumed, following Hashin’s idea, that uniform biaxial compressive strength is far larger than the uniaxial compressive strength [9]. That is

(29) $$\frac{Y_{C}}{Y_{\mathrm{Cbi}}}\approx 0$$

Substituting Equation (29) into Equations (27) and (28) obtains

(30) $$F_{2}^{\mathrm{mc}}=-\frac{1}{Y_{C}}+\frac{Y_{C}}{4S_{T}^{2}}$$

(31) $$F_{22}^{\mathrm{mc}}=\frac{1}{4S_{T}^{2}}$$

A uniform tri-axial stress state of compression ($\sigma_{11}=\sigma_{22}=\sigma_{33}=-\sigma^{*}$) is used here to determine the expression of $F_{12}^{\mathrm{mc}}$. This approach is similar to the one proposed to gain the value of coefficient $F_{12}^{\mathrm{fc}}$ (see in Section 2.1). $F_{12}^{\mathrm{mc}}$ would be approximated as

(32) $$F_{12}^{\mathrm{mc}}=\frac{F_{44}^{\mathrm{mc}}-4F_{22}^{\mathrm{mc}}}{2}=0$$

Regarding MFT, utilizing Equation (20) to transverse tensile strength $Y_{T}$ renders

(33) $$F_{22}^{\mathrm{mt}}Y_{T}^{2}+F_{2}^{\mathrm{mt}}Y_{T}=1$$

Failure data for combined stresses are needed to provide a necessary additional equation, as the transverse tensile test information provides only one equation for the determination of the two coefficients $F_{22}^{\mathrm{mt}}$ and $F_{2}^{\mathrm{mt}}$. Alternatively, to avoid using extra experiments that are difficult to be carried out, a feasible equation is constructed by expecting that the failure envelope surface is completely smooth.

Hence a smooth condition of the failure envelope is employed as

(34) $$\nabla F_{\mathrm{MFT}}|{}_{I_{2}=0}=\nabla F_{\mathrm{MFC}}|{}_{I_{2}=0}$$

where $\nabla F_{\mathrm{MFT}}$ and $\nabla F_{\mathrm{MFC}}$ are the gradients of the functions for MFT and MFC failure. The above equation means that the slope of the tangent of the MFT function is equal to that for MFC at $I_{2}=0$. Finally, Equation (34) gives

(35) $$F_{2}^{\mathrm{mt}}=F_{2}^{\mathrm{mc}}=-\frac{1}{Y_{C}}+\frac{Y_{C}}{4S_{T}^{2}}$$

Meanwhile $F_{22}^{\mathrm{mt}}$ could be estimated by

(36) $$F_{22}^{\mathrm{mt}}=\frac{1}{Y_{T}^{2}}+\frac{1}{Y_{T}Y_{C}}-\frac{Y_{C}}{4Y_{T}S_{T}^{2}}$$

Combining Equation (23) and Equation (24) results in

(37) $$c_{\mathrm{mt}}=c_{\mathrm{mc}}$$

and $F_{12}^{\mathrm{mt}}$ must satisfy

(38) $$F_{12}^{\mathrm{mt}}=\frac{c_{\mathrm{mc}}}{c_{\mathrm{mt}}}F_{12}^{\mathrm{mc}}=F_{12}^{\mathrm{mc}}=0$$

For both $F_{12}^{\mathrm{mt}}$ and $F_{12}^{\mathrm{mc}}$, their values of zero may indicate a physical phenomenon that the Poisson’s effect due to $\sigma_{11}$ does not influence the occurrence of MFT and MFC.

Now the failure mode-independent criterion derived on strain energy can be summarized for UD composites.

- Fiber tension failure $\sigma_{11}\geq 0$
  (39) $$\frac{\sigma_{11}^{2}}{X_{T}^{2}}-\frac{\sigma_{11}\left(\sigma_{22}+\sigma_{33}\right)}{2X_{T}^{2}}=1$$
- Fiber compression failure $\sigma_{11}<0$
  (40) $$\frac{\sigma_{11}^{2}}{X_{C}^{2}}-\frac{\sigma_{11}\left(\sigma_{22}+\sigma_{33}\right)}{2X_{C}^{2}}=1$$
- Matrix tension failure $\sigma_{22}+\sigma_{33}\geq 0$
  (41) $$\begin{array}{l} \left(\frac{1}{Y_{T}^{2}}+\frac{1}{Y_{T}Y_{C}}-\frac{Y_{C}}{4Y_{T}S_{T}^{2}}\right){\left(\sigma_{22}+\sigma_{33}\right)}^{2}+\left(-\frac{1}{Y_{C}}+\frac{Y_{C}}{4S_{T}^{2}}\right)\left(\sigma_{22}+\sigma_{33}\right)\\ +\frac{1}{S_{T}^{2}}\left(\tau_{23}^{2}-\sigma_{22}\sigma_{33}\right)+\frac{1}{S_{L}^{2}}\left(\tau_{12}^{2}+\tau_{13}^{2}\right)=1 \end{array}$$
- Matrix compression failure $\sigma_{22}+\sigma_{33}<0$
  (42) $$\begin{array}{l} \frac{1}{4S_{T}^{2}}{\left(\sigma_{22}+\sigma_{33}\right)}^{2}+\left(-\frac{1}{Y_{C}}+\frac{Y_{C}}{4S_{T}^{2}}\right)\left(\sigma_{22}+\sigma_{33}\right)+\frac{1}{S_{T}^{2}}\left(\tau_{23}^{2}-\sigma_{22}\sigma_{33}\right)\\ +\frac{1}{S_{L}^{2}}\left(\tau_{12}^{2}+\tau_{13}^{2}\right)=1 \end{array}$$

## 3. Failure Mode-Interactive Criterion Based on Strain Energy Release

Following the previous statement, the strain energy related to fiber failure, $U_{e}^{f}$, has no impact on matrix damages. This hypothesis is consistent with the one adopted by most of the existing criteria, e.g., Hashin’s criterion [9] or Puck’s criterion [8], which treats fiber fracture independent of matrix cracking because their potential fracture planes are perpendicular. However, the weakening effect of $\sigma_{11}$ on the transverse mechanical behavior has been clearly observed by experimental evidence [24]. In other words, the predictions gained by mode-independent criteria are not conservative in some loading conditions, such as combined $\sigma_{11}-\sigma_{22}$. From the microscopic point of view, the reason may be that the existence of local fiber-matrix debonding and matrix micro-cracks will weaken the ability of composites to resist both fiber- and matrix-dominated failure. However, if the focus is shifted to describe above failure mode interactions on the lamina level, the existing methods introduce artificial parameters that are hardly or even impossibly measured, e.g., portions of the failure function proposed by Cuntze [17] and degradation factors used by Puck et al. [13,24]. In this paper, a method is proposed below on a rational physical basis to eliminate empiricism, as far as failure mode interactions of UD composites are concerned.

Obviously, both fiber failure and matrix failure in materials correspond to strain energy release. As a consequence, if an interaction of fiber and matrix failure has to be executed, the strain energy that causes ‘mixed’ material failure should physically involve two independent parts, i.e., the one resulting in matrix failure and that causing failure, as discussed in previous Section 2. The strain energies released at the macro level have already considered the effect of complicated and uncertain factors (e.g., defects) at the microscale. The failure criterion can be given as

(43) $$\alpha^{ij}U_{e}^{i}+\beta^{{}^{ij}}U_{e}^{j}=G_{c}^{ij}\left(I_{1},I_{2}\right)\;\;\left(i=\mathrm{ft},\mathrm{fc}\;j=\mathrm{mt},\mathrm{mc}\right)$$

where $\alpha^{ij}$ and $\beta^{ij}$ represent the efforts of fiber-related and matrix related strain energy to the mixed failure, respectively. Superscripts i and j denote specific failure mode. $G_{c}^{ij}$ is the critical strain energy for the corresponding failure mode, and can be derived by using a second-order approximation of $I_{2}$ with consideration of different effects of the hydrostatic stress ($I_{1}$ and $I_{2}$) on the mechanical behavior of UD composites. Substituting Equations (11) and (19) into Equation (43) obtains

(44) $$\begin{array}{l} \alpha^{ij}\frac{S_{11}}{2}\sigma_{11}^{2}+\left(\alpha^{ij}+\beta^{ij}\right)S_{12}\sigma_{11}\left(\sigma_{22}+\sigma_{33}\right)+\beta^{ij}\frac{S_{22}}{2}{\left(\sigma_{22}+\sigma_{33}\right)}^{2}+\\ \beta^{ij}\frac{S_{44}}{2}\left(\tau_{23}^{2}-\sigma_{22}\sigma_{33}\right)+\beta^{ij}\frac{S_{66}}{2}\left(\tau_{12}^{2}+\tau_{13}^{2}\right)\\ =a_{ij}{\left(\sigma_{22}+\sigma_{33}\right)}^{2}+b_{ij}\left(\sigma_{22}+\sigma_{33}\right)+c_{ij} \end{array}$$

Rearranging the terms gives

(45) $$\begin{array}{l} F_{11}^{ij}\sigma_{11}^{2}+F_{12}^{ij}\sigma_{11}\left(\sigma_{22}+\sigma_{33}\right)+F_{22}^{ij}{\left(\sigma_{22}+\sigma_{33}\right)}^{2}+F_{2}^{ij}\left(\sigma_{22}+\sigma_{33}\right)+\\ F_{44}^{ij}\left(\tau_{23}^{2}-\sigma_{22}\sigma_{33}\right)+F_{66}^{ij}\left(\tau_{12}^{2}+\tau_{13}^{2}\right)=1 \end{array}$$

Considering the available uniaxial test information in Equation (45) and using the similar approaches discussed in Section 2.1 and Section 2.2, all the coefficients except $F_{12}^{ij}$ are equal to those of the corresponding stress terms appearing in Equations (39)–(42). The coefficient $F_{12}^{ij}$ describes only the interaction between direct stresses $\sigma_{11}$, $\sigma_{22}$ and $\sigma_{33}$, indicating that a tri-axial stress state having an independent $\sigma_{11}$ in addition to $\sigma_{22}=\sigma_{33}$ can deduce the analytical expression of $F_{12}^{ij}$. First assuming a tri-axial compressive stress state $\sigma_{11}=\sigma_{22}=\sigma_{33}=-\sigma^{*}<0$ in Equation (45),

(46) $$\left(F_{11}^{\mathrm{fcmc}}+4F_{22}^{\mathrm{fcmc}}+2F_{12}^{\mathrm{fcmc}}-F_{44}^{\mathrm{fcmc}}\right){\left(\sigma^{*}\right)}^{2}-2F_{2}^{\mathrm{fcmc}}\sigma^{*}=1$$

It is anticipated that the material would sustain a stress level significantly higher, which can be treated as infinite for mathematical convenience, than its uniaxial compressive strength. This can be re-written into

(47) $$\begin{array}{cc} F_{12}^{\mathrm{fcmc}} & =\frac{1}{2{\left(\sigma^{*}\right)}^{2}}+\frac{F_{2}^{\mathrm{fcmc}}}{\sigma^{*}}+\frac{\left(F_{44}^{\mathrm{fcmc}}-F_{11}^{\mathrm{fcmc}}-4F_{22}^{\mathrm{fcmc}}\right)}{2}\\ & \approx -\frac{F_{11}^{\mathrm{fcmc}}}{2}=-\frac{1}{2X_{C}^{2}} \end{array}$$

The failure in UD composites may be solely presented as FFT mode with respect to the stress condition of $\sigma_{11}\geq 0$ and $\sigma_{22}=\sigma_{33}=-\sigma^{*}<0$, due to the assumption that the MFC mode is not activated under uniform biaxial compression (see in Equation(29)). According to the equivalence of Equation (12) and Equation (45),

(48) $$F_{11}^{\mathrm{ftmc}}X_{T}^{2}-2F_{12}^{\mathrm{ftmc}}X_{T}\sigma^{*}+\left(4F_{22}^{\mathrm{ftmc}}-F_{44}^{\mathrm{ftmc}}\right){\left(\sigma^{*}\right)}^{2}-2F_{2}^{\mathrm{ftmc}}\sigma^{*}=F_{11}^{\mathrm{ft}}X_{T}^{2}-2F_{12}^{\mathrm{ft}}X_{T}\sigma^{*}$$

Utilizing the condition of $\sigma^{*}$ at failure is extremely high, $F_{12}^{\mathrm{ftmc}}$ is approximated as

(49) $$F_{12}^{\mathrm{ftmc}}=F_{12}^{\mathrm{ft}}-\frac{F_{2}^{\mathrm{ftmc}}}{X_{T}}=-\frac{1}{2X_{T}^{2}}+\frac{1}{X_{T}Y_{C}}-\frac{Y_{C}}{4X_{T}S_{T}^{2}}$$

Triaxial tensile test data for the determination of coefficients $F_{12}^{\mathrm{ftmt}}$ are considered. But since available data is limited in the current literature and experimental methods are immature, an alternative method is given to smooth the failure envelope at the point $\sigma_{22}+\sigma_{33}=0$. Hence,

(50) $$F_{12}^{\mathrm{ftmt}}=F_{12}^{\mathrm{ftmc}}$$

Likewise, the application of the smooth condition, it gives

(51) $$F_{12}^{\mathrm{fcmt}}=F_{12}^{\mathrm{fcmc}}$$

So far, the failure mode-interactive criterion has been proposed, through a logical-mathematical derivation regarding physical circumstances of strain energy release, as

- FFT and MFT $\sigma_{11}\geq 0$ and $\sigma_{22}+\sigma_{33}\geq 0$
  (52) $$\begin{array}{l} \frac{\sigma_{11}^{2}}{X_{T}^{2}}+\left(-\frac{1}{2X_{T}^{2}}+\frac{1}{X_{T}Y_{C}}-\frac{Y_{C}}{4X_{T}S_{T}^{2}}\right)\sigma_{11}\left(\sigma_{22}+\sigma_{33}\right)+\left(\frac{1}{Y_{T}^{2}}+\frac{1}{Y_{T}Y_{C}}-\frac{Y_{C}}{4Y_{T}S_{T}^{2}}\right){\left(\sigma_{22}+\sigma_{33}\right)}^{2}\\ +\left(-\frac{1}{Y_{C}}+\frac{Y_{C}}{4S_{T}^{2}}\right)\left(\sigma_{22}+\sigma_{33}\right)+\frac{1}{S_{T}^{2}}\left(\tau_{23}^{2}-\sigma_{22}\sigma_{33}\right)+\frac{1}{S_{L}^{2}}\left(\tau_{12}^{2}+\tau_{13}^{2}\right)=1 \end{array}$$
- FFC and MFT $\sigma_{11}<0$ and $\sigma_{22}+\sigma_{33}\geq 0$
  (53) $$\begin{array}{l} \frac{\sigma_{11}^{2}}{X_{C}^{2}}-\frac{\sigma_{11}\left(\sigma_{22}+\sigma_{33}\right)}{2X_{C}^{2}}+\left(\frac{1}{Y_{T}^{2}}+\frac{1}{Y_{T}Y_{C}}-\frac{Y_{C}}{4Y_{T}S_{T}^{2}}\right){\left(\sigma_{22}+\sigma_{33}\right)}^{2}\\ +\left(-\frac{1}{Y_{C}}+\frac{Y_{C}}{4S_{T}^{2}}\right)\left(\sigma_{22}+\sigma_{33}\right)+\frac{1}{S_{T}^{2}}\left(\tau_{23}^{2}-\sigma_{22}\sigma_{33}\right)+\frac{1}{S_{L}^{2}}\left(\tau_{12}^{2}+\tau_{13}^{2}\right)=1 \end{array}$$
- FFT and MFC $\sigma_{11}\geq 0$ and $\sigma_{22}+\sigma_{33}<0$
  (54) $$\begin{array}{l} \frac{\sigma_{11}^{2}}{X_{T}^{2}}+\left(-\frac{1}{2X_{T}^{2}}+\frac{1}{X_{T}Y_{C}}-\frac{Y_{C}}{4X_{T}S_{T}^{2}}\right)\sigma_{11}\left(\sigma_{22}+\sigma_{33}\right)+\frac{1}{4S_{T}^{2}}{\left(\sigma_{22}+\sigma_{33}\right)}^{2}\\ +\left(-\frac{1}{Y_{C}}+\frac{Y_{C}}{4S_{T}^{2}}\right)\left(\sigma_{22}+\sigma_{33}\right)+\frac{1}{S_{T}^{2}}\left(\tau_{23}^{2}-\sigma_{22}\sigma_{33}\right)+\frac{1}{S_{L}^{2}}\left(\tau_{12}^{2}+\tau_{13}^{2}\right)=1 \end{array}$$
- FFC and MFC $\sigma_{11}<0$ and $\sigma_{22}+\sigma_{33}<0$
  (55) $$\begin{array}{l} \frac{\sigma_{11}^{2}}{X_{C}^{2}}-\frac{\sigma_{11}\left(\sigma_{22}+\sigma_{33}\right)}{2X_{C}^{2}}+\frac{1}{4S_{T}^{2}}{\left(\sigma_{22}+\sigma_{33}\right)}^{2}+\left(-\frac{1}{Y_{C}}+\frac{Y_{C}}{4S_{T}^{2}}\right)\left(\sigma_{22}+\sigma_{33}\right)\\ +\frac{1}{S_{T}^{2}}\left(\tau_{23}^{2}-\sigma_{22}\sigma_{33}\right)+\frac{1}{S_{L}^{2}}\left(\tau_{12}^{2}+\tau_{13}^{2}\right)=1 \end{array}$$

## 4. Validation Studies

Experimental measurements from World-Wide Failure Exercise (WWFE), organized by Hinton, Soden and Kaddour [32,33], are used here to evaluate the capability of the two proposed failure criteria. The material properties are collected in Table 1. The Hashin criterion, which is admired for its simplicity of concept and wide incorporation into FEA commercial codes, is also adopted to make a parallel comparison.

Honestly, we should admit that there could be possibilities to achieve the wrong results in the experiments. The evaluations obtained by direct comparisons with experimental results of a high dispersion are certainly irrational. However, because a large number of experimental data from WWFE have been used by a wide range of popular failure theories to estimate their predictability, as a result, the reliability of experimental results from WWFE has been demonstrated by many researchers, so it may be feasible to validate the proposed theory by using these results. In addition, in contrast to the failure criteria that employ input parameters hardly determined by the existing test methods, for our failure criteria, the input parameters, which should be provided as known conditions, are solely conventional uniaxial strengths of the composite lamina. These strengths can be measured by mature test methods, and corresponding test standards are formed, such as American Society for Testing and Materials (ASTM) standards. Thus, it helps avoid experimental error or control high dispersions regarding with input parameters, resulting in high fidelity of predictions given by our criteria.

### 4.1. $\sigma_{22}$ − $\tau_{12}$ Failure Envelopes

**Figure 1:**
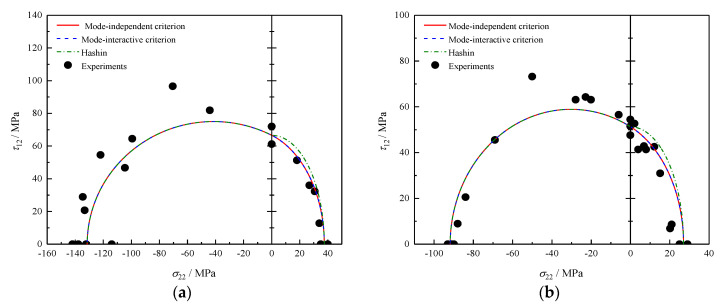
Failure envelopes and experimental results under $\sigma_{22}-\tau_{12}$ for (**a**) E-glass/ LY556 and (**b**) AS4/55A.

Figure 1 shows the predicted $\sigma_{22}-\tau_{12}$ failure envelopes for two different materials: E-Glass/LY556 [32] and AS4/55A [35]. It is observed that both the mode-independent criterion and the mode-interactive criterion coincide exactly with each other and fit well the experimental results obtained. For the stress interval of $\sigma_{22}<0$, when the lamina is subjected to moderate transverse compressive stress $\sigma_{22}$, the phenomenon that higher shear stress ($\tau_{12}>S_{L}$) could be sustained without fracture is successfully obtained. Better agreements between predicted and measured results are achieved than those given by the Hashin criterion in the case of $\sigma_{22}\geq 0$. The difference is a logical consequence of whether the influence of hydrostatic stress on the critical strain energy. The Hashin criterion overestimates the capacity of UD composites against MFT fracture, resulting in non-conservative predictions. Besides, both proposed criteria eliminate the undesired sharp corner existing at $\sigma_{22}=0$.

### 4.2. $\sigma_{11}$ − $\sigma_{22}$ Failure Envelopes

**Figure 2:**
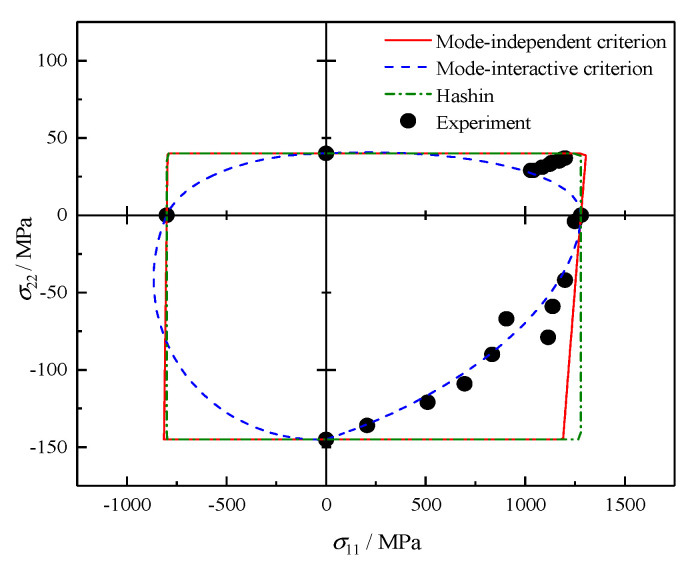
Failure envelopes and experimental results under $\sigma_{11}-\sigma_{22}$ for E-glass/MY750.

Soden and Hinton [32] tested E-glass/MY750 lamina subjected to longitudinal and transverse loading. The experimental data and the theoretical results for combined $\sigma_{11}$−$\sigma_{22}$ are presented in Figure 2. The Hashin criterion degenerates into the maximum stress criterion, and the shape of its failure envelope is a rectangle with the boundaries corresponding to basic tensile and compressive strengths. A slight difference between failure envelopes of the mode-independent criterion and Hashin criterion is observed and may be caused by the Poisson’s effect of longitudinal and transverse directions according to Equation (11). However, the quadrilateral lines, indicating the independence of two stress components $\sigma_{11}$ and $\sigma_{22}$, is not supported by the test data. In contrast, a relatively better agreement between experiments and theoretical results, particularly in the tension-compression quadrants, once on account for the interaction between fiber and matrix failure. It is worth mentioning that in the compression–compression quadrant, the biaxial strength is predicted to exceed the uniaxial longitudinal compressive strength by a maximum of 8% at the stress ratio $\sigma_{11}/\sigma_{22}=19.2$.

### 4.3. $\sigma_{11}$ − $\tau_{12}$ Failure Envelopes

**Figure 3:**
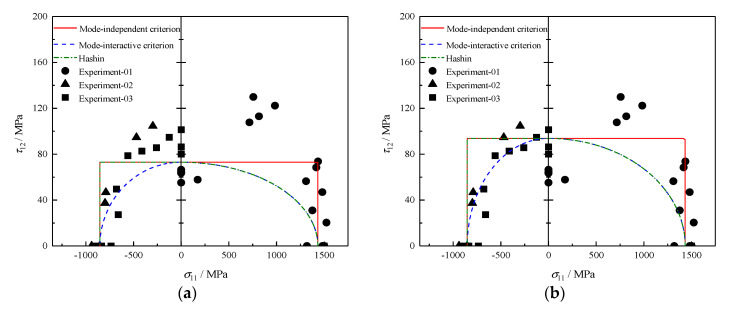
Failure envelopes and experimental results under $\sigma_{11}-\tau_{12}$ for T300/BSL914C with (**a**) $S_{L}=73\mathrm{MPa}$ and (**b**) $S_{L}=93.8\mathrm{MPa}$.

The predicted $\sigma_{11}-\tau_{12}$ failure envelopes of T300/BSL914C carbon epoxy [32] are plotted in Figure 3. A high dispersion is found for test data, especially for that from Experiment-01. These experimental errors, e.g., the average value of longitudinal shear strength $S_{L}=73\mathrm{MPa}$, would affect some theoretical results, as shown in Figure 3a. But if we abandon the data from Experiment-01 due to significant data scatter, the accuracy of predictions could be improved with $S_{L}=93.8\mathrm{MPa}$. The failure envelope given by the mode-independent criterion degenerates into a rectangle, meaning that $\sigma_{11}$ and $\tau_{12}$ control failure in fiber and matrix modes, respectively. The mode-interactive criterion is, compared to the other criteria, more conservative in predicting fracture for the $\sigma_{11}-\tau_{12}$ diagram. At the same time, it achieves the best agreement with the measurements. It should be noted that even though the envelope of Hashin criterion stays consistent with the one gained by the mode-interactive criterion when $\sigma_{11}\geq 0$, their physical interpretation underlying is distinctly different. Hashin’s theory asserts the failure mode is still FFT failure even under extremely small $\sigma_{11}$, which is inconceivable because any fiber may not be ruptured from a microscopic point of view. The proposed mode-interactive criterion, on the contrary, provides a reasonable explanation that the potential failure mode will gradually change from fiber failure into matrix failure with the decrease of $\sigma_{11}$, which will always keep making contributions to failure regardless of failure modes.

### 4.4. Off-Axis Tension Failure

**Figure 4:**
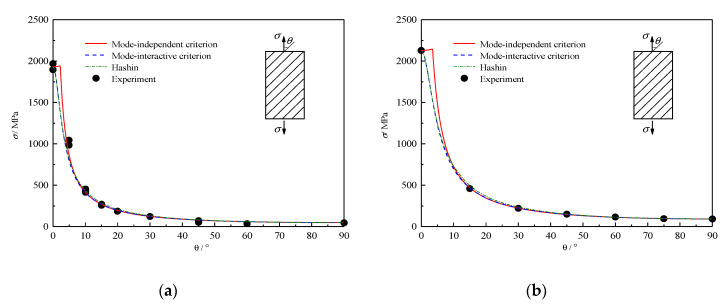
Comparison between the predicted off-axial tensile strengths and experimental results for (**a**) T800H/2500EP and (**b**) AS4/PEEK.

Figure 4 shows the experimental and predicted relations between the off-axis angle and the peak tensile stress. Test data for materials AS4/PEEK and T800H/2500EP are reported from references [36,37]. All theories fit the experimental results well. The predicted failure mode transition angles from FFT to MFT are listed in Table 2. It is mentioned that the inherent mode predicted by the mode-interactive criterion may be estimated by comparing values of FF-related function and MF-related function. As MFT fracture can be observed in the off-axis tension experiments if the loading angle exceeds 5°, the mode transition angles predicted by both proposed criteria are more reasonable than the Hashin criterion.

### 4.5. $\sigma_{22}$ − $\sigma_{33}$ (with $\sigma_{11}=\sigma_{33}$) Failure Envelopes

**Figure 5:**
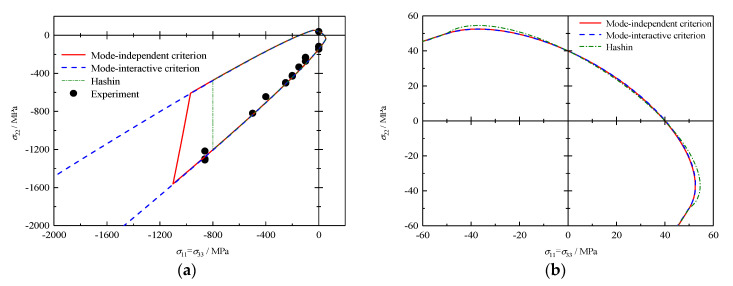
Failure envelopes and experimental results under $\sigma_{22}-\sigma_{33}$ (where $\sigma_{11}=\sigma_{33}$) for E-Glass/MY750: (**a**) whole envelope and (**b**) partial enlargement.

The predictions of the failure envelopes for E-glass/MY750 lamina [33] loaded with varied combinations of $\sigma_{22}$ and $\sigma_{11}=\sigma_{33}$ are presented in Figure 5. MF fracture is predicted as the primary failure mode by all failure theories in this stress state. In the quadrant of tri-axial compression, both envelopes provided by Hashin’s and the mode-independent criteria are cut off by FF fracture, as shown in Figure 5a, while the envelope of the proposed mode-interactive criteria is open due to the adopted assumption stating infinite tri-axial compressive strength in mathematics. In fact, in a real test, failure could be initiated by a local defect. The comparison between all failure theories and experiments shows an exceptionally good agreement. Partial enlargement in Figure 5b demonstrates that the proposed criteria provide relatively more conservative results and a smoother surface than the Hashin criterion.

### 4.6. $\tau_{12}$ − $\sigma_{22}$ (with $\sigma_{11}=\sigma_{22}=\sigma_{33}$) Failure Envelopes

**Figure 6:**
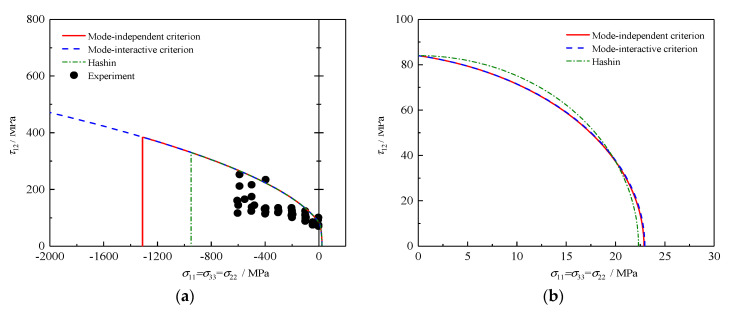
Failure envelopes and experimental results under $\tau_{12}-\sigma_{22}$ (where $\sigma_{11}=\sigma_{22}=\sigma_{33}$) for T300/PR39: (**a**) whole envelope and (**b**) partial enlargement.

Figure 6 presents a comparison between the predictions and test data under combined hydrostatic pressure and shear stress for a composite lamina made of T300/PR39 [33]. In the tri-axial compressive regime, an enhancement in shear strength is observed from Figure 6a. With increasing compressive hydrostatic stress, the failure envelopes of the mode-independent criterion and Hashin criterion are cut off by a vertical line, suggesting that the failure mode eventually changes to fiber fracture. In contrast, the openness of the failure envelope can be seen for the proposed mode-interactive criterion, due to the underlying manipulation of infinite strengths under hydrostatic pressure. MFT failure is predicted by all failure theories in the tensile regime, as shown in Figure 6b, and the present criteria provide relatively more conservative results at high shear stress. Nevertheless, all theories significantly overestimate the effect of hydrostatic pressure on strength of the composite lamina. The possible reason may be the hypothesis that the ratio of uniaxial compressive strength to biaxial compressive strength is approximately zero (see in Equation (29)), resulting in an overestimation of the coefficient $F_{2}^{ij}$ related to the linear stress term of $\left(\sigma_{22}+\sigma_{33}\right)$ mathematically.

### 4.7. $\sigma_{11}$ − $\sigma_{22}$ (with $\sigma_{22}=\sigma_{33}$) Failure Envelopes

**Figure 7:**
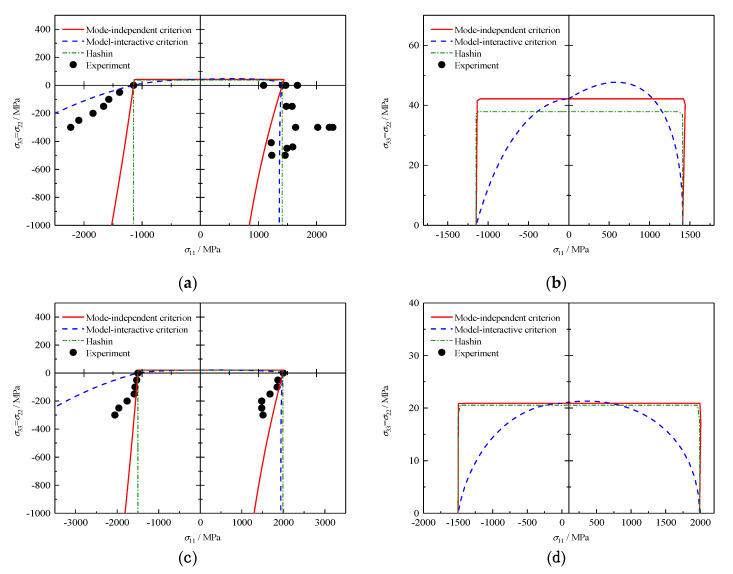
Failure envelopes and experimental results under $\sigma_{11}-\sigma_{22}$ (where $\sigma_{22}=\sigma_{33}$) for S-glass/epoxy: (**a**) whole envelope and (**b**) partial enlargement, and for A-S carbon/epoxy: (**c**) whole envelope and (**d**) partial enlargement.

The last cases are related to composite materials S-glass/epoxy and A-S carbon/epoxy [33]. The failure envelopes under varied combinations of $\sigma_{11}$ and $\sigma_{22}=\sigma_{33}$ are plotted in Figure 7. All theories predict the failure envelopes are open in the presence of transverse pressures. In the negative $\sigma_{22}=\sigma_{33}$ regime, the loci obtained by Hashin’s fibre fracture (FF) criterion, which degenerates into the maximum stress criterion, are two infinitely extended vertical lines. Meanwhile, the loci obtained by the mode-independent criterion are not vertical due to the extra consideration of a Poisson’s effect of transverse stresses. In the region characterized by tri-axial compression, it is evident that the proposed failure mode-interactive criterion exhibits a better predictive ability, particularly for S-glass/epoxy, despite the overestimation of infinite bi-axial and tri-axial compressive strengths. Noted that in practice, composite materials will rupture due to their imperfect micro-structures, e.g., manufacturing defects. Thus, results predicted by the criterion considering the interaction effect are acceptable for the reason of lack of accessible multi-axial experimental data. The distribution of data in the tension–compression quadrant explains the reasonability that regarding FFT fracture as the dominating failure mode for all analyzed criteria.

## 5. Concluding Remarks

In the present study, two new strain energy-based failure criteria, respectively referring to mode-independent and mode-interactive criteria, are proposed and evaluated for fiber-reinforced composite materials. All coefficients employed in the formulation of the theory are obtained by following logical deductions from a set of predefined assumptions. On the premise of the lack of experimentally feasible multi-axial strengths, the assumption of infinite strength under bi-axial and tri-axial compressive stress provides a condition for determining coefficients of the terms representing stress interaction. The main conclusions are listed here.

(1) From the viewpoint of energy release, it demonstrates that the general expression for fiber fracture mode should not involve the shear stress term due to a mathematical contradiction in the formulation of failure theory. A genuine feature of physics purely from the mathematical and logical deduction is explained. In the proposed theory, despite the work is based on strain energy density, the specific value of energy is not required to be determined. Experimental data show that the inclusion of the shear stress term in the Hashin FFT criterion leads to the underestimation of failure strength.

(2) The employment of linear stress terms is a logical outcome considering the different influences of hydrostatic stress on longitudinal and transverse elastic moduli. The hydrostatic stress may not result directly in macroscopic failure, but it could affect the strain energy density stored in composite materials. The well-known phenomenon that moderate transverse compression impedes shear fracture could not be predicted if abandoning the hydrostatic stress effect.

(3) A failure criterion at the lamina level is further proposed for characterizing microscopic interactions between matrix-dominated and fiber-dominated failure modes. The interactive coefficients, which represent the coupling effects of dominant stresses driving FF and MF are determined under certain tri-axial stress states.

(4) Experimental verification has shown that both the present criteria, especially the mode-interactive criterion, work reasonably well for predicting the failure of most UD-laminates under biaxial, off-axis and tri-axial loading. The input parameters are limited to the conventional uniaxial tensile, compressive and shear strengths, and no empirical or artificially defined input parameters are required to calibrate. Thus the proposed criteria have a wide range of applicability and can be incorporated into finite element (FE) codes in a relatively easy manner.

## Figures and Tables

**Table 1:** Strength properties of composites [34].

Material	*X*_T_/MPa	*X*_C_/MPa	*Y*_T_/MPa	*Y*_C_/MPa	*S*_L_/MPa	*S*_T_/MPa
E-Glass/LY556	1140	570	37.5 *	131.5 *	66.6 *	40
AS4/55A			27	91.8	51.3	26.8
E-glass/MY750	1280 *	800 *	40 *	145 *	73	50
T300/BSL914C	1433.6 *	853 *	27	200	73 *	41
AS4/PEEK	2128	954.6	93	205.9	133	72.7
T800H/2500EP	1934	-	48.5	120	77.8	40
S-glass/epoxy	1410 *	1147 *	63	180	72	50
A-S carbon/epoxy	2000 *	1500	38	150	72	40
T300/PR319	1378	950	40	125	76 *	45

* Average value.

**Table 2:** Mode transition angles induced by the off-axial load.

Failure Criterion	Mode Transition Angle/°	
	T800H/2500EP	AS4/PEEK
Mode-independent criterion	2.2	3.4
Mode-interactive criterion	2.2	3.4
Hashin	8.9	11.8
